# Research on the Coordinate Attention Mechanism Fuse in a YOLOv5 Deep Learning Detector for the SAR Ship Detection Task

**DOI:** 10.3390/s22093370

**Published:** 2022-04-28

**Authors:** Fang Xie, Baojun Lin, Yingchun Liu

**Affiliations:** 1 Aerospace Information Research Institute, Chinese Academy of Sciences, Beijing 100094, China; xief@microsate.com; 2 School of Optoelectronics, University of Chinese Academy of Sciences, Beijing 100094, China; liuyc@microsate.com; 3 Innovation Academy for Microsatellites, Chinese Academy of Sciences, Shanghai 201210, China; 4 Shanghai Engineering Center for Microsatellites, Shanghai 201304, China; 5 School of Information Science and Technology, ShanghaiTech University, Shanghai 201210, China

**Keywords:** synthetic aperture radar, ship object detection, coordinate attention mechanism, YOLOv5, onboard computing

## Abstract

The real-time performance of ship detection is an important index in the marine remote sensing detection task. Due to the computing resources on the satellite being limited by the solar array size and the radiation-resistant electronic components, information extraction tasks are usually implemented after the image is transmitted to the ground. However, in recent years, the one-stage based target detector such as the You Only Look Once Version 5 (YOLOv5) deep learning framework shows powerful performance while being lightweight, and it provides an implementation scheme for on-orbit reasoning to shorten the time delay of ship detention. Optimizing the lightweight model has important research significance for SAR image onboard processing. In this paper, we studied the fusion problem of two lightweight models which are the Coordinate Attention (CA) mechanism module and the YOLOv5 detector. We propose a novel lightweight end-to-end object detection framework fused with a CA module in the backbone of a suitable position: YOLO Coordinate Attention SAR Ship (YOLO-CASS), for the SAR ship target detection task. The experimental results on the SSDD synthetic aperture radar (SAR) remote sensing imagery indicate that our method shows significant gains in both efficiency and performance, and it has the potential to be developed into onboard processing in the SAR satellite platform. The techniques we explored provide a solution to improve the performance of the lightweight deep learning-based object detection framework.

## 1. Introduction

With the self-illumination capability, the Synthetic Aperture Radar (SAR) provides images independent of the weather and illumination conditions and has been widely applied to plant observation, especially in marine monitoring [1,2]. Ship object detection from SAR images is widely applied in both civilian and military marine monitoring tasks such as illegal smuggling, port management, and military reconnaissance. Nowadays, with the flourish of the commercialization of aerospace activities, Low Earth Orbit (LEO) satellite launch missions show a remarkable rapid increasing trend [3]. Meanwhile, the LEO-SAR satellite also has the trend of low cost, miniaturization, and constellation. Under this trend, numerous SAR images are generated on-orbit, bringing a new challenge for the ship object detection task; meanwhile, ship detection in SAR images has become a hot spot.

### 1.1. Traditional Detector

Traditional methods for SAR ship detection tasks mainly used the constant false alarm rate (CFAR) detector [4], and the CFAR method determines the detection threshold according to the pre-established clutter statistical model. However, as a predefined model based on sea clutter modeling features to find bright pixels, it is vulnerable to the influence of ocean current and climate, leading to its application scenarios being very limited. The template-based method [5] is another common method, templates are designed manually depending on expert experience, and every template corresponds to ship features (length, width, perimeter, area, contour, texture, etc.), by sliding the template window to detect the object. Using this method on SAR images with large scenes would take a huge volume of computing. In addition, because of the backscatter imaging mechanism, features extracted from SAR images are highly sensitive to the SAR acquisition geometry [6], building an accurate ship detection and classification model was almost impossible, leading to the template-based method’s generalization ability being poor [7].

### 1.2. Deep Learning-Based Detector

Fortunately, the breakthrough of convolutional neural network (CNN) based deep learning technology [8] provides a new ability for object detection. The CNN has a receptive field similar to the human eye [9], which can observe the target information more comprehensively, so it has been widely used in the field of object detection.

The CNN-based object detection method with powerful feature representation capabilities started to come into the mainstream. CNN-based object detectors are usually categorized into two-stage detectors and one-stage detectors. Two-stage detectors include a Region-based convolutional network (R-CNN) [10] series, Fast region-based convolutional (fast R-CNN) [11], Faster region-based convolutional network (Faster R-CNN) [12], etc. The region-based method generates independent region proposals firstly, then extracts object feature vectors from each region, and then uses a classifier such as a linear support vector machine (SVM) to classify the objects. R-CNN is time-consuming because the training stages are divided into multiple stages to perform a convolution forward pass for each object proposal without sharing computation [11]. Fast-R-CNN reduces the computational complexity by using the Spatial Pyramid Pooling networks (SPP nets) [13] to speed up R-CNN by sharing computation and using a soft-max function instead of SVMs to improve the performance of R-CNNs. Faster R-CNN [12] introduced a novel Region Proposal Networks (RPNs) to replace the typical region proposal methods with state-of-the-art object detection networks. The two-stage method usually has a large number of model parameters, and the consumption of computing resources is very high. One-stage detectors do not include a region proposal layer in the head model, with run detection directly over a dense sampling of locations, such as Retina Net [14], Single Shot Multi-Box Detector (SSD) [15], and You Only Look Once (YOLO) series detectors [16,17,18,19,20]. Those detectors are designed for optical Internet images, aiming at the particularity of ship detection tasks in SAR images. Some researchers have started to improve one-stage detectors to solve the SAR ship detection problem in recent years. In 2019, Wang et al. [21] proposed an Improved Retina Net-based ship detection technique for Gaofen-3 SAR data. Gang et al. [22] proposed an N-YOLO architecture, by enhancing the contrast between target and background to improve the accuracy. Those impressive improvements reveal that one-stage detectors can efficiently detect multi-scale ships with a high detection accuracy in SAR data. Table 1 shows the related work discussion on DL-based one-stage ship detectors in recent years.

In this paper, we study the problem of efficiency optimization of the YOLOv5 detector in the SAR ship detection task. Our work focuses on three main points, the first point is that with the need for on-board processing, and we choose the lightweight YOLOv5 as the optimized baseline. The second point is that YOLOv5 is designed based on the Internet image dataset, but SAR images have large-scale changes in feature and noise interference different from internet images. Some studies have shown that the CBAM attention mechanism [26] can effectively help YOLOv5 improve performance in remote sensing images [27]. We choose the more lightweight Coordinate Attention (CA) Mechanism [28] to study the problem of CA mechanism fusion with the baseline method. The third point is that YOLOv5 is designed as a multi-class detector, but the ship detection task in SAR images is a binary classification problem. We investigate whether there is still an optimization margin for the YOLOv5 backbone network.

In view of the above three research points, we propose an optimized SAR ship detection framework: You Only Look Once with Coordinate Attention for SAR ship detection (YOLO-CASS), and we test it on SSDD [29] SAR ship dataset. The experimental results show that YOLO-CASS has lightweight, good robustness ability. The main contributions of this paper are as follows:

1. For the task of ship detection in SAR images, choosing a reasonable position to integrate the coordinate attention mechanism into the backbone network can effectively improve the detection performance, robustness, and anti-noise detection capabilities.
2. Reducing the number of network layers in the backbone network reasonably has little impact on the SAR ship detection task.
3. The YOLO-CASS model achieves 97.8 mAP@IOU = 0.5, which is only 1.81 MB. In addition, we have evaluated the training energy consumption. The training energy cost of YOLO-CASS is only 14% of YOLOv5X. This lightweight model offers possibilities and engineering guidance for porting to satellite-borne computing for on-orbit interpretation and online training,

The rest of this paper is organized as follows: the second chapter provides a brief review and related theoretical basis, including the yolov5 detection framework and coordinate attention mechanism. Then, the third chapter provides the introduction of the experimental dataset and the evaluation methods, and the experimental results. The fourth chapter provides visual demonstrations of the experimental and discussions of the test results. Finally, the conclusions are drawn and the related future work is given.

## 2. Methodology

### 2.1. Background of YOLOv5

**Figure 1:**
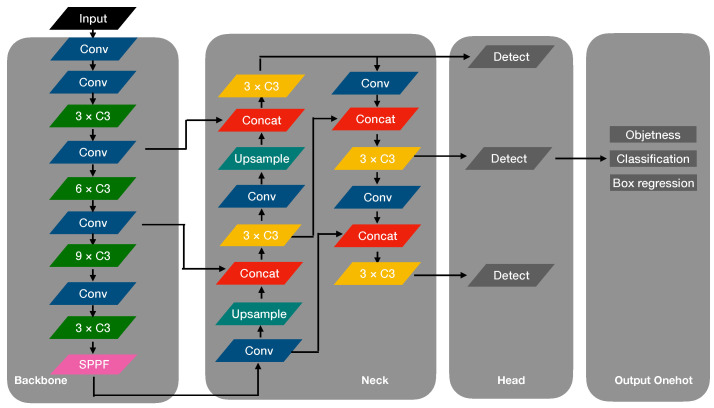
The network structure of YOLOv5 deep learning framework. It is mainly composed by a backbone network, neck network. and head network.

YOLOv5 is a very popular deep learning framework. The main idea of YOLO is to divide the input image into K×K cell grid [16]. If the center of the object falls into one grid cell, the grid cell is set as responsible for predicting the object. YOLOv5 regards the detection task as a regression problem by using a single neural network to predict the bounding box and classes directly. Figure 1 shows the last structure of the YOLOv5 (6.0 edition) deep learning framework. The whole network is composed of three parts: backbone, neck, and head. The input image adopts Mosaic data augmentation [20], adaptive anchor, and adaptive image scaling then enters the backbone. The backbone network features an extract layer with multi-deep convolution layers for input images. The neck network is a feature aggregation layer between the head and backbone that collects as much multi-scale information extracted by the backbone as possible before it is fed to the head. The head network uses a YOLOv3 [18] detect head with CIOU [30] loss function for output multi-scale object information including location, classification, and bounding box regression.

**Figure 2:**
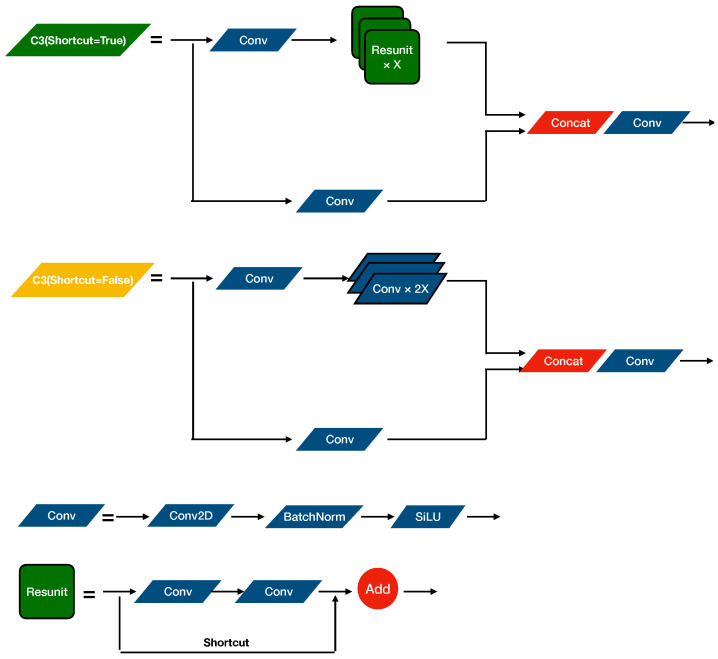
The structure of the CONV unit module and C3 unit module. The green C3 unit contains multiple Resunit with shortcut structures applied in the backbone network. The yellow C3 unit without a shortcut structure is applied in the neck network. The Conv2D refers to 2D convolution. The BachNorm refers to batch normalization.

As shown in Figure 1, the backbone network is composed of CONV units, C3 units, and an SPPF unit. Figure 2 shows the structure of those units. The CONV unit performs convolution calculation, batch normalization, and through a Sigmoid-weighted Linear Unit (SiLU) activation function in sequence. The SiLU activation function is defined as the following equation:

(1) $$\mathrm{SiLU}\left(x\right)=x\cdot \sigma \left(x\right),\;\mathrm{where}\;\sigma \left(z\right)={\left(1+\exp\left(-z\right)\right)}^{-1}$$

SiLU is a special case of swish function [31], due to avoiding the issue of the ReLU function that easily causes neuron death in the training process. SiLU can be loosely viewed as a smooth function that nonlinearly interpolates between the linear function and the ReLU function [32].

**Figure 3:**
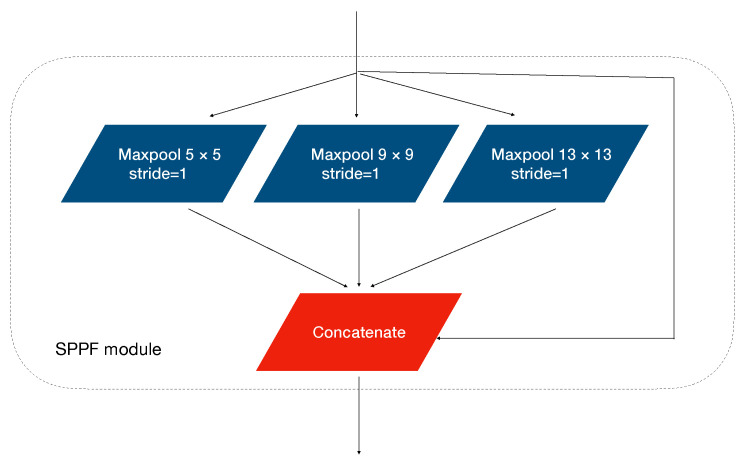
The SPPF module: it divides the input tensors into four channels, performs maximum pooling operations respectively, and then fuses the information output through the concatenate operation.

The green C3 module in Figure 2 shows the C3 module with a shortcut structure, and it is applied in the backbone network. The C3 module is a Cross Stage Partial (CSP) structure, it equally divides the input tensor into two branches and performs convolution operations, respectively: one branch passes through a CONV module and then passes through multiple Resunit [33], which is a residual structure to avoid degradation problems in the deep compute process. The other branch convolutes directly and then concatenates the two branches and operates a CONV module. The yellow C3 module in the neck is different from C3 in the backbone. The difference is that C3 in the neck replaced the Resunit with multiple CONV modules without residual structure. Figure 3 shows the Spatial Pyramid Pooling Fusion (SPPF) module [34], and it concatenates four different fixed block pooling operations to realize feature fusion for different scale receptive fields to adapt complex multi-target images and to improve the receptive field of the network.

The loss function of YOLOv5 consists of three parts: bounding box loss, object confidence loss, and classification loss. The ship detection task is a binary classification problem; thus, we do not need to consider the classification loss function. Object confidence is used for reflecting the level of confidence that the bounding box contains an object. Formula (2) denotes the confidence probabilities that each grid cell. The confidence of the bounding box actually indicates whether there is an object center point at this grid. The closer the confidence predicted value is equal to 1, the more likely there is an object in this bounding box and vice versa. The confidence can be defined as follows:

(2) $$\begin{array}{cc} \; & C\left(Object\right)=\mathrm{Pr}\left(\;\mathrm{ShipObject}\;\right)\times \;\mathrm{IOU}{\;}_{pred}^{truth} \end{array}$$

where $\;\mathrm{IOU}{\;}_{pred}^{truth}$ measures the correlation between ground truth and prediction bounding box. The $\;\mathrm{IOU}{\;}_{pred}^{truth}$ indices is defined as the following equation:

(3) $$\;\mathrm{IOU}{\;}_{pred}^{truth}=\frac{B_{pred}\cap B_{groundtruth}}{B_{pred}\cup B_{groundtruth}}$$

where *B*_*pred*_ is the area of the predicted bounding box, and *B*_*grountruth*_ is the area of ground truth. To evaluate the loss of confidential information, the confidence loss is the binary cross-entropy between the prediction and the ground truth, which can be defined as follows:

(4) $$\begin{array}{c} L_{\mathrm{conf}}=-\lambda_{pos}\sum_{i=0}^{K^{2}}\sum_{j=0}^{B}I_{i,j}^{obj}\left[\hat{C_{i}^{j}}\log\left(C_{i}^{j}\right)+\left(1-{\hat{C_{i}}}^{j}\right)\log\left(1-C_{i}^{j}\right)\right]-\\ \lambda_{neg}\sum_{i=0}^{K^{2}}\sum_{j=0}^{B}I_{i,j}^{obj}\left[\hat{C_{i}^{j}}\log\left(C_{i}^{j}\right)+\left(1-{C_{i}}^{j}\right)\log\left(1-\hat{C_{i}^{j}}\right)\right] \end{array}$$

where $K^{2}$ denotes the number of grids, and *B* denotes the number of bounding boxes in each grid, $I_{i,j}^{obj}$ denotes that the *j*th bounding box in the *i*th cell is responsible for the prediction, when an object exists in a bounding box, $I_{i,j}^{obj}$ is equal to 1; otherwise, it is 0. $\hat{C_{i}^{j}}$ represents the prediction confidence of the *j*th bounding box in the *i*th grid, and $C_{i}^{j}$ represents the ground truth confidence of the *j*th bounding box in the *i*th grid, $\lambda_{pos}$ is used to increase the loss from bounding box coordinate predictions and $\lambda_{neg}$ is to decrease the loss from confident predictions for boxes that do not contain objects, respectively.

### 2.2. Coordinate Attention Mechanism

The attention mechanism originated from the study of human vision. In cognitive science, due to bottlenecks in information processing, humans selectively focus on a portion of all information while ignoring other visible information. The above mechanism is often referred to as the attention mechanism. Different parts of the human retina have different degrees of information processing ability, namely acuity, and only the fovea has the strongest acuity. In order to reasonably utilize the limited visual information processing resources, humans need to select a specific part of the visual area and then focus on it. The attention mechanisms have been proven to be helpful in a variety of computer vision tasks [35]. The most popular attention mechanism is Squeeze and Excitation (SE) attention [28]. This mechanism was first applied to mobile networks, it calculates channel attention with the help of 2D global pooling, and provides tableless performance improvements with a low computational cost. Channel attention uses global pooling to encode spatial information globally. However, it compresses global spatial information into channel descriptors, so it is difficult to preserve location information that is crucial for capturing spatial structure in vision tasks; therefore, a CA mechanism is proposed. The coordinate attention mechanism takes into account both inter-channel relationships and positional information.

**Figure 4:**
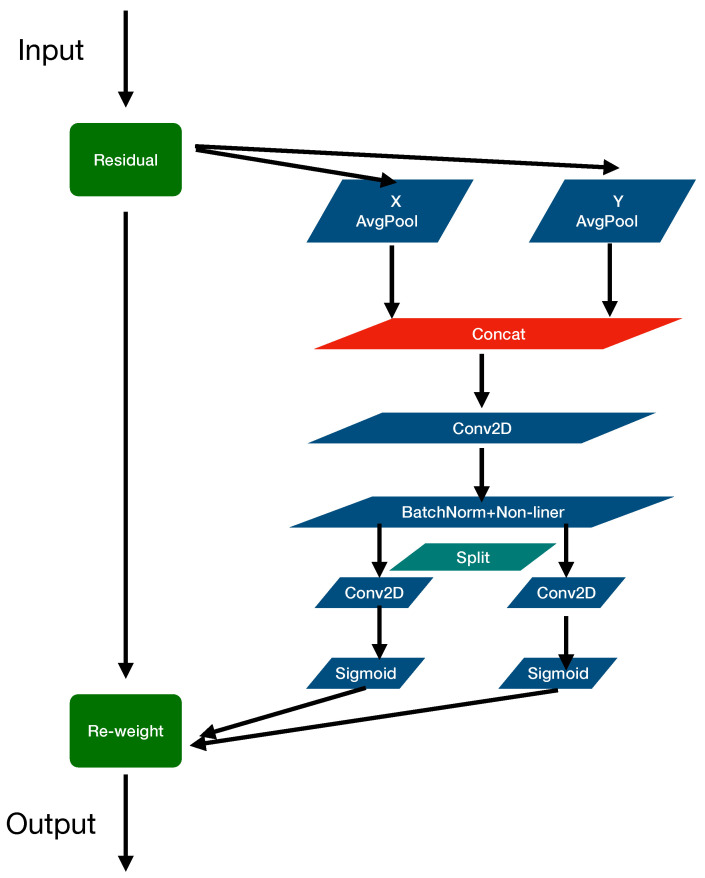
The coordinate attention block, ‘X Avg Pool’ and ‘Y Avg Pool’ refer to 1D horizontal global pooling and 1D vertical global pooling.

Figure 4 shows the composition block diagram of the CA mechanism, and the detailed principle of the coordinate attention mechanism can be referred to in the paper [28], which proves that the coordinate attention mechanism performs much better than other attention mechanisms [26,36,37] with the lightweight property. The coordinate attention mechanism improves the efficiency of information flow in the neural network, due to helping the neural network pay attention to valid coordinates and suppress invalid coordinates.

## 3. Experimental

### 3.1. Dataset

**Figure 5:**
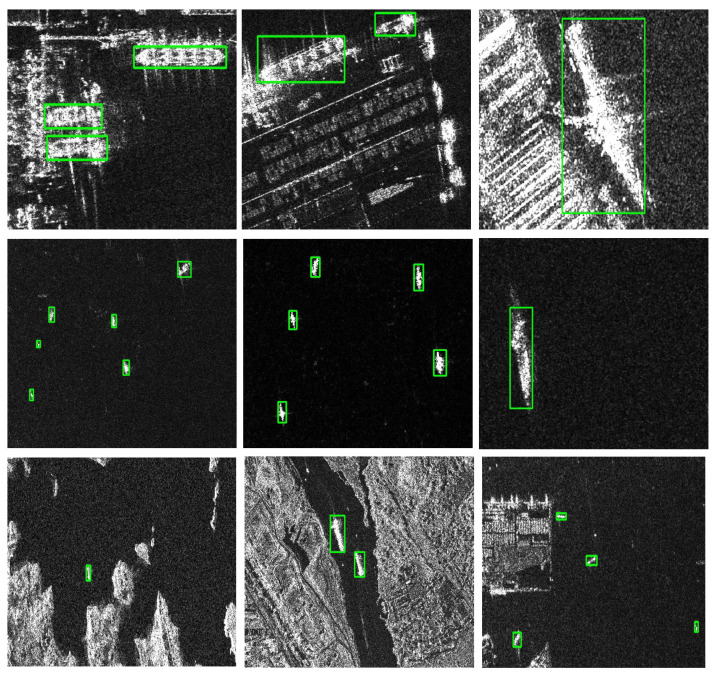
Sample images from the SAR ship detection dataset (SSDD) dataset. The SSDD dataset contains ships in different scenes: The first row shows the scene where the ship is inshore, the second row shows the scene where the ship is offshore, and the third row shows a complex scene with a large field of view.

In this work, the SAR Ship Detection Dataset (SSDD) [29] is used as a benchmark dataset for model evaluation. In SSDD, there are a total of 1160 images and 2456 ships. The average number of ships per image is 2.12. We divide the dataset into two parts: the training set, and the test set, with ratios of 928 images and 232 images, respectively. Figure 5 shows examples from the dataset.

**Figure 6:**
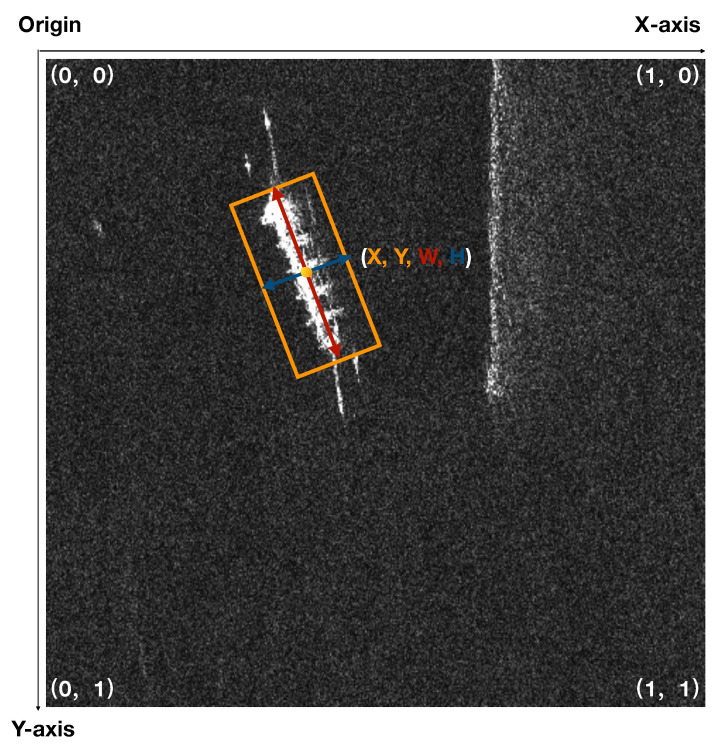
The annotation information of a labeled ship chip. Each ship label in the dataset is represented by a rectangular bounding box, each bounding box contains four dimensions of information, and it uses the normalized bounding box center point (x, y) and the length and width of the rectangle (w, h).

**Figure 7:**
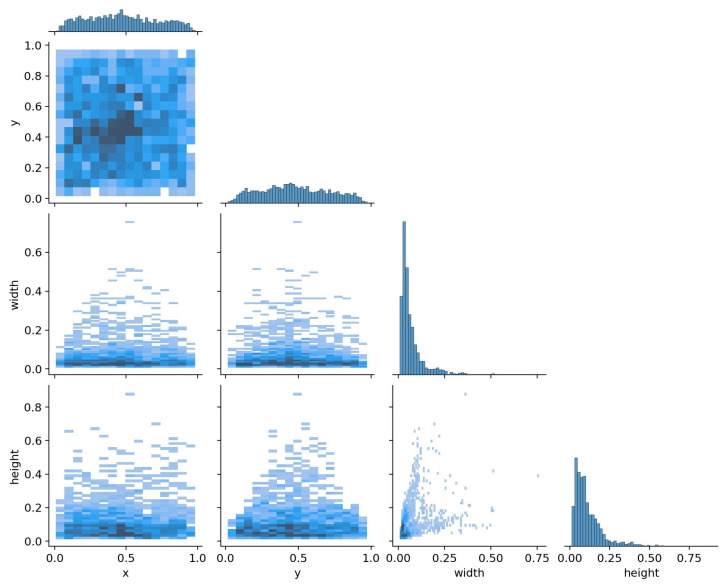
The location distribution of the dataset instances (*x*- and *y*-values of the center point), and the correlation distribution of the width and height of the ground true bounding box.

In the dataset, the labels’ files are in line with the PASCAL VOC standard. We convert it to YOLO format. These parameters describe bounding boxes which are described in normalized format (from 0 to 1) as shown in Figure 6, and Figure 7 shows the target distribution of the SSDD data dataset. The ship targets in the dataset are all labeled, and each image chip corresponds to a txt file including three pieces of information:

1. Ship identification marks;
2. Ship center position: x center, y center;
3. Bounding box measurement: width, height.

### 3.2. Evaluation Methods

To evaluate the performance of the model, those four indicators are dedicated to evaluating the experimental results: Precision, Recall, and Mean Average Precision (mAP).

For one object detection test, if the model predicts a target and the IOU is larger than a threshold, the test results regard it as a true positive (TP). If the model predicts that there is a target in an image but actually the image does not contain the target, the test result is regarded as a false positive (FP). Conversely, if the model predicts that there is no target but actually the image contains the target, the test result is regarded as a false negative (FN). If the model predicts that there is no target and the image actually does not contain the target, the result is regarded as a true negative (TN).

Precision rate describes how many positive examples predicted by the classifiers are real positive examples. Precision refers to the proportion of ground truth ships predicted by networks in all predictions. The equation defines the precision ratio:

(5) $$\;\mathrm{Precision}\;=\frac{TP}{TP+FP}$$

Recall rate describes how many real positive examples in the test set are selected by the classifiers from the perspective of real results. Recall refers to the proportion of ground truth ships predicted by networks in all ground truth ships. The equation defines the recall ratio:

(6) $$\;\mathrm{Recall}\;=\frac{TP}{TP+FN}$$

In order to characterize the comprehensive performance of the model in precision and recall, the current precision and recall can be calculated each time by gradually reducing the IOU threshold. Taking recall as the horizontal axis and precision as the vertical axis, we can obtain the precision–recall (*P–R*) curve. Formula (7) defines the mean average precision, and it describes the area under *P–R* curves to illustrate the comprehensive performance of the different models:

(7) $$\mathrm{mAP}=\int_{0}^{0}P\left(R\right)dR$$

All experiments are implemented with an Nvidia 2070s GPU. The operating system is Ubuntu 16.04, the integrated development environment (IDE) is PyCharm, and the deep learning platform is PyTorch.

### 3.3. Experiment #1: Fuse Configuration Variation

In this experiment, we study the performance analysis of the coordinate attention mechanism fused with YOLOv5. YOLOv5 provides five different scales for their model N, S, M, L, and X which stand for Nano, Small, Medium, Large, and X large, respectively. Each of these scales applies a different multiplier to the depth and width of the model, meaning the size and complexity of each model are scaled, but the structure remains constant.

**Figure 8:**
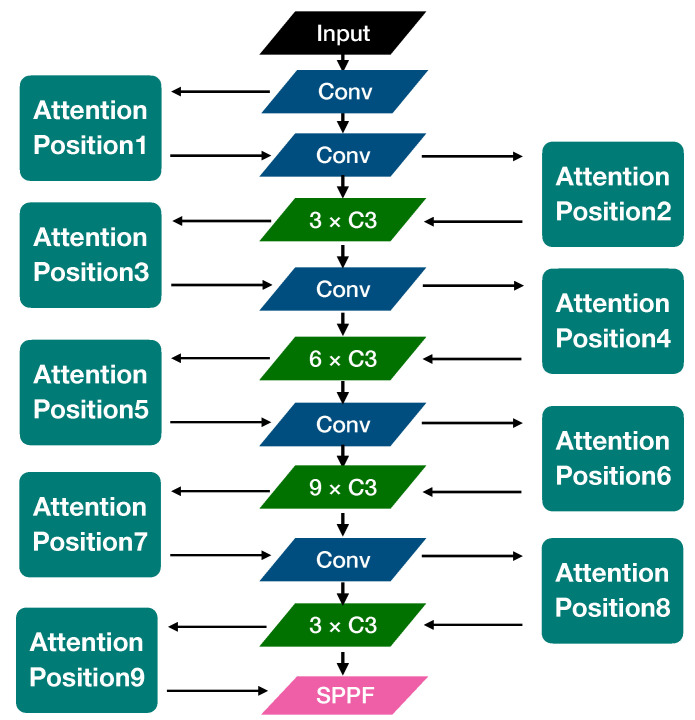
The corresponding map of the position relationship added by the Attention Mechanism in the Backbone. For example, if the CA mechanism is fused at Position 1, then the fusion network is named YOLOv5 + CA(1).

This paper aims to study lightweight algorithms. Therefore, we study the fusion efficiency of the CA mechanism in YOLOv5n. As shown in Figure 8, we select nine reserved fusion point positions in the backbone of YOLOv5n, add the CA module, and then train the model and test its performance separately. The hyperparameters are set as follows: the training steps are 300 epochs; warmup epoch and warmup momentum are respectively set as 3 and 0.8; the training and test batch size is 16. The optimization algorithm is an SGD optimizer with an initial learning rate of 0.01; the momentum and weight decay are respectively set as 0.937 and 0.0005.

The statistics of the experimental results are listed in Table 2. From this experiment, since the scores of mAP_0.5_ are all overly similar, we use the score value of mAP_0.5:0.95_ to evaluate the performance of each model after fusion. We found that different locations have different effects on model accuracy. The best position is Position 7 with 65.8 mAP_0.5:0.95_, and the worst position is Position 4 with 63.7 mAP_0.5:0.95_.

**Figure 9:**
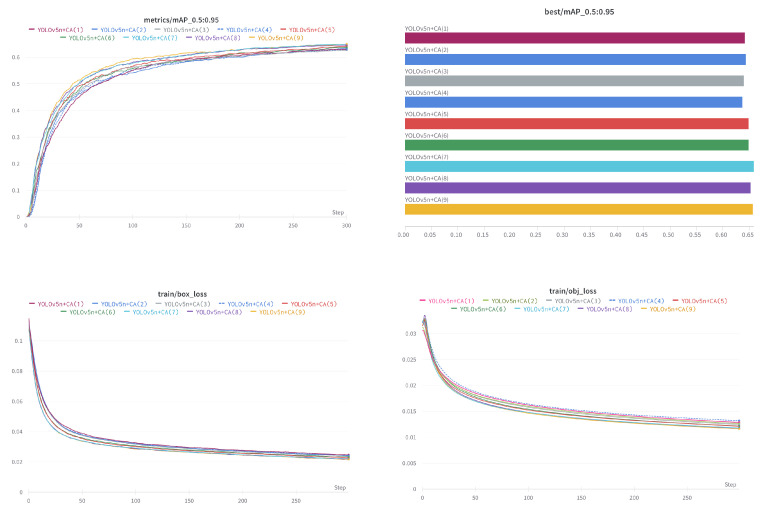
Characteristic curves of different fusion configurations. The upper left subgraph shows the change of mAP scores of different models with the training epoch. The upper right subgraph shows the maximum mAP distribution of each model in 300 epochs. The lower left and lower right subgraphs show the bounding box loss and the confidential loss in the training process, respectively. The curves are smoothed for more readability.

We have plotted mAP-Epoch and Loss-Epoch curves in Figure 9. We can find that deploying the attention module at the depth of the Backbone (close to the Neck network) can effectively speed up the training convergence rate and improve the model performance (by analyzing the Loss–Epoch curve, and the best mAP score), but it does not mean that the more the deeper the better, the largest performance gain is shown at Position 7. When the CA module is deployed deeper than Position 7, the performance begins to degrade.

Through the experiments in this section, we found that adding the CA module to the middle-deep position in the backbone can speed up the convergence speed during training and improve the mAP score on the SSDD dataset. Position 7 obtains the best value, which is higher than Position 4 of 2.1%.

### 3.4. Experiment #2: Scale and Layer Redundancy Variation

Based on the above analysis, in this experiment, we use YOLOv5n + CA(7) as the benchmark to study the influence of different layers in the network and different model scales on the performance. We take YOLOv5n + CA(7) as the baseline and adjust the weight factor of C3 modules in the backbone from (3, 6, 9, 3) to (3, 6, 6, 3) in turn, and we call this model YOLOv5n + CA(7)-reduce.

**Figure 10:**
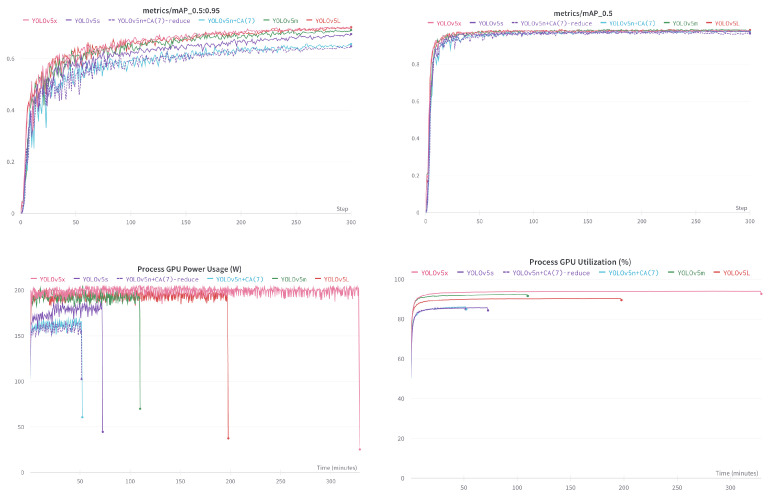
The upper left and upper right subplots give the scores of different models at IOU = 0.5:0.95 and IOU = 0.5, and the lower left and lower right subplots give the process GPU power usage and process GPU utilization of different models, respectively.

Then, we separately train and compare those models. Due to the limitation of GPU memory, the batch size of this experiment is set to 4; other parameter settings are the same as in experiment 1. Table 3 shows the experimental results, and Figure 10 shows the mAP-epoch curves.

As Figure 10 shows, obviously, although the mAP performance of the simplified YOLOv5n + CA(7)reduce is 0.7% lower than the benchmark, it is only 0.2% lower in mAP_0.5_, and the model parameters are 5% less than the benchmark. That said, the lightweight network should still have optimization margins. Compared with other larger-scale models, although mAP has decreased, the degree of lightness of the model is more significant than the decrease in inaccuracy. The smallest YOLOv5n + CA(7)reduce is only 2% of the parameter volume of the best performing YOLOv5X model. Moreover, the small model in the training process is more energy-saving and environmentally friendly. The energy consumption of training once YOLOv5n + CA(7) only lasts 52 minutes at 180 w, compared with YOLOv5X, which lasts 329 minutes at 200 w, saving 86% of energy consumption.

## 4. Discussion

In this paper, we investigate the performance of the attention mechanism fuse in different positions in the YOLOv5 backbone, on the SAR ship detection task. The experimental results show that the attention mechanism is fused at different positions in the backbone network, and the performance gap of the models is obvious.

**Figure 11:**
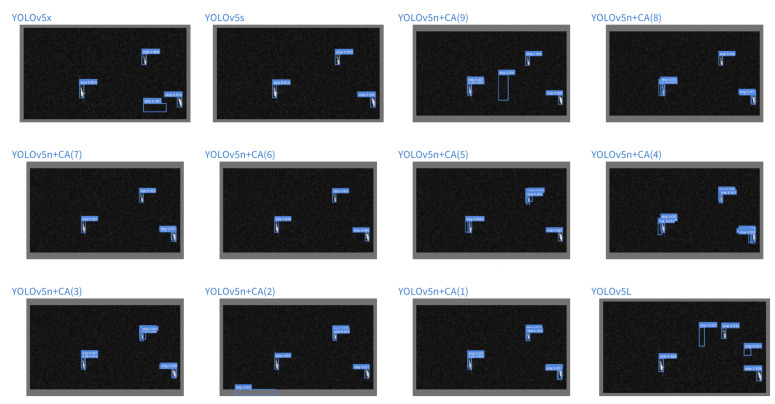
Visualization of a typical offshore scenario during the testing process for experiment. Each subgraph represents the Bbox generation of the scene by different models before NMS.

**Figure 12:**
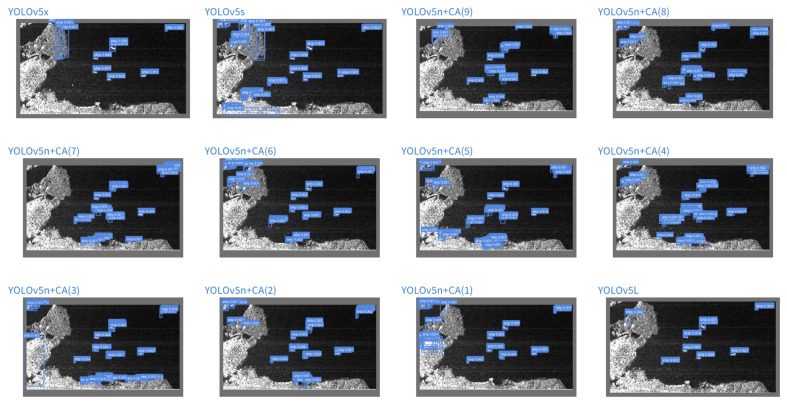
Visualization of a typical inshore scenario during the testing process for experiment. Each subgraph represents the Bbox generation of the scene by different models before NMS.

We debug the Bounding-box generated before NMS. Here, we select two typical visual experiment scenarios to discuss the reasons for the improvement. Figure 11 shows an SAR image scene of the offshore ships. By observing the performance of each model in the experiment, we can clearly see from the model that fusion with the attention mechanism could suppress false alarms caused by background noise effectively. Figure 12 shows a complex inshore scene. From this part, it can be seen that the gain brought by increasing the coordinate attention mechanism is more obvious; especially, the CA module is integrated into position (7), which is significantly reduced. The false alarm phenomenon is due to the interference of complex features.

By analyzing the visualization results, the inshore scenes with large-scale target gaps, since the ship is the object of interest, and the land is not the object of interest, the CA mechanism can help the backbone to effectively pay attention to the ship, thereby suppressing other unimportant information in the image, which shows that a reasonable setting of the CA mechanism can effectively improve the robustness of the model to scale changes. In addition, for offshore ships, the backbone network with the CA mechanism can effectively filter out the interference of noise, which shows that the reasonable setting of the CA mechanism can effectively improve the robustness of the model to the influence of noise. This is especially suitable for SAR remote sensing images with native noise. In addition, since the CA mechanism is very lightweight, it has little effect on the volume increase of the model. By adding the CA module to the YOLOv5n, the model size is only 1.81 M. Therefore, the lightweight YOLOv5 method integrates the coordinate attention mechanism is suitable for the SAR ship detection task. We call this type of method You Only Look Once with Coordinate Attention for SAR Ship detection (YOLO-CASS).

We try to further simplify the parameters of the YOLO-CASS model. In Experiment 2, we found that the effect of model scale on accuracy is far less than that of scale variation, which we believe is due to the fact that SAR images have only one channel and a simple gray-background ratio compared to other optical image datasets, feature comparison single, so it can obtain better feature extraction effects without a complex backbone network model. Therefore, we believe that the backbone network can be further streamlined, which will be one of our future works. In addition, YOLO-CASS is particularly suitable for deployment on satellites with limited computing power due to its lightweight characteristics. Port YOLO-CASS to aerospace computing platforms should be another topic for future research.

## 5. Conclusions

The real-time detection of ships is an important indicator in the task of marine remote sensing detection. Because computing resources on satellites are limited by the limited energy system power and radiation-hardened electronics, information extraction tasks are typically performed after the images are transmitted to the ground. In this paper, we studied the performance of different fusion configurations of a coordinate attention mechanism fuse in the YOLOv5 backbone, and proposed an end-to-end SAR ship detection framework—YOLO-CASS. YOLO-CASS integrates a lightweight coordinate attention mechanism. The performance comparison with the baseline method shows that YOLO-CASS has an effective information extraction ability and is energy-efficient. Due to the lightweight characteristics of YOLO-CASS, it has great potential to be transplanted on the onboard computer to realize the on-orbit ship detection task on the SAR satellite.

Additionally, this paper aims to study the efficiency of different fusion configurations, which is a relative performance comparison. Therefore, the model uses the same initial weights and does not implement pre-train [38] for each special network branch in this paper. Inspired by XAI [39,40] technology, exploring the interpretability of the YOLO-CASS framework will provide more detailed and rich information for the specialized ship detection network architecture design. It will be an important work in the future to improve the performance of the model and further reduce the number of parameters by pre-training of each single-modality branch and fine-tuning the entire network.

## Figures and Tables

**Table 1:** The related work discussion on DL-based ship detectors.

Paper	Technique	Work Goals
2019 Wang et al. [21]	RetinaNet	mAP improvement
2021 Yao Chen et al. [23]	DarkNet-53	mAP improvement
2021 Gang Tang et al. [22]	YOLO	mAP improvement
2022 Xu, Pan, et al. [24]	CFAR&YOLOv4	mAP Improvement
2022 Xu, Xiaowo et al. [25]	YOLOv5	Efficiency Optimization
This paper	YOLOv5&CA	Efficiency Optimization

**Table 2:** Performance scores of models with different fusion configurations on the SSDD dataset.

Method	Precision(%)	Recall(%)	mAP_0.5_ (%)	mAP_0.5:0.95_(%)
YOLOv5n + CA(1)	95.3	93.6	97.2	64.2
YOLOv5n + CA(2)	95.7	94.1	97.9	64.4
YOLOv5n + CA(3)	98.0	91.6	96.8	64.1
YOLOv5n + CA(4)	95.5	93.4	96.9	63.7
YOLOv5n + CA(5)	97.2	94.9	98.0	64.8
YOLOv5n + CA(6)	96.4	92.7	97.7	64.8
YOLOv5n + CA(7)	95.6	95.6	97.8	65.8
YOLOv5n + CA(8)	96.3	94.5	96.3	65.3
YOLOv5n + CA(9)	95.6	94.9	97.0	65.6

**Table 3:** Comparison of performance scores of YOLOv5 models with different layers and scales.

Method	Precision (%)	Recall (%)	mAP_0.5_ (%)	mAP_0.5:0.95_ (%)	Model Size (Byte)	Training Time (min)
YOLOv5x	98.3	95.2	98.0	72.4	86.2 M	328.92
YOLOv5l	98.0	96.0	98.4	72.3	46.1 M	199.51
YOLOv5m	97.6	96.5	98.4	71.0	20.9 M	141.41
YOLOv5s	96.0	96.2	97.8	69.6	7.00 M	76.63
YOLOv5n + CA(7)	95.6	95.6	97.8	65.8	1.81 M	52.20
YOLOv5n + CA(7)-reduce	95.5	93.4	97.6	65.1	1.72M	50.30

## Data Availability

Not applicable.

## Abbreviations

The following abbreviations are used in this manuscript:

CFAR	Constant False Alarm Rate
CNN	Convolutional Neural Network
CBAM	Convolutional Block Attention Module
CA	Coordinate Attention
DL	Deep Learning
FP	False Positive
FN	False Negative
GPU	Graphics Processing Unit
IOU	Intersection over Union
IDE	Integrated Development Environment
LEO	Low Earth Orbit
mAP	mean Average Precision
NMS	Non Maximum Suppression
R-CNN	Region-based Convolutional Network
RPN	Region Proposal Networks
ReLU	Linear Rectification Function
SVM	Support Vector Machine
SPP	Spatial Pyramid Pooling
SiLU	Sigmoid-weightedLinear Unit
SAR	Synthetic Aperture Radar
SSD	Single Shot Multi-Box Detector
SSDD	SAR Ship Detection Dataset
SE	Squeeze and Excitation
TP	True Positive
TN	True Negative
YOLO	You Only Look Once
XAI	eXplainable Artificial Intelligence
